# Enlargements of the Spleen—III

**Published:** 1908-08-08

**Authors:** 


### Medicine.

### ENLARGEMENTS OF THE SPLEEN—III.

### TUMOURS THAT MAY BE MISTAKEN FOR AN ENLARGED SPLEEN (*continued*).

Suprarenal Growths.

Malignant disease of the suprarenal gland has been known to cause a large tumour, difficult to distinguish not only from a splenic, but also from a renal tumour. As a result of the close proximity of the suprarenal body to the kidney, and of the liability of the latter to become infiltrated by growth of the former, a condition which may lead to hæmaturia, and thus complicate the diagnosis still further, the physical signs of a tumour are practically the same in both viscera.

Two points of difference which may sometimes be of service are that suprarenal tumours may push the colon downwards instead of forwards, and that in children an abnormal development of hair and of the genitals is to be observed in some cases of adrenal neoplasm.

Splenic Flexure Growths.

New growths, nearly always malignant, of the splenic flexure of the colon are usually annular in form, in which case they do not give rise to any definite tumour, the chief symptoms being those of intestinal obstruction. Occasionally, however, the growth may be more extensive and diffuse, with the result that a fairly large tumour is found to occupy the left hypochondrium. The points which help in distinguishing this from an enlarged spleen are the following:—

(*a*) A growth of the splenic flexure of the colon may be found to shift its position a little from day to day, or even during a physical examination, unless it is quite fixed by adhesions.

(*b*) It is resonant on percussion in front.

(*c*) It has no well-defined edge nor notch.

(*d*) It is usually associated with signs of intestinal trouble, such as alternating constipation and diarrhœa or hæmorrhage.

(*e*) There may be secondary deposits elsewhere. Blood examination is not always helpful. The only splenic tumours diagnosable by the blood-count are those associated with leuchæmia, pernicious anæmia, malaria, and typhoid fever. In malignant disease of the colon there is likely to be anæmia of the secondary type.

Pancreatic Tumours.

On occasions an enlarged spleen may be mistaken for a pancreatic tumour, particularly a pancreatic cyst. The points of distinction are briefly as follows:—

(*a*) A pancreatic tumour is usually situated more in the median line of the abdomen, between the ensiform cartilage and the umbilicus. In the case of very large cysts, however, the mass may fill the greater part of the abdominal cavity.

(*b*) On account of its close proximity to the aorta, a well-marked transmitted pulsation may be seen and felt in the tumour; this pulsation diminishes but little when the patient assumes the knee-elbow position, whereas a spleen, by falling away from the aorta, ceases to transmit pulsation in this position.

(*c*) No definite edge or notch can be felt.

(*d*) The stomach may lie in front of the upper part of the tumour; if this point cannot be determined by an ordinary physical examination of the abdomen, the stomach may be distended with gas by giving a Seidlitz powder in separate halves.

(*e*) A splenic tumour very rarely extends to the right of the median line of the abdomen above the umbilicus unless the enlargement is very great, in which case the lower pole of the spleen would be some inches below the level of the umbilicus.

The following case illustrates the difficulties which may arise in distinguishing between a pancreatic and a splenic tumour. The case was thought to be one of pancreatic cyst; exploratory laparotomy was performed; a large spleen was found, and the diagnosis was changed to that of splenic anæmia.

The patient was a lad, aged nineteen, who came under observation for anæmia and weakness. His father died of phthisis and alcoholism. The patient had suffered from measles when a child; had sustained a severe kick in the abdomen from a horse ten years ago; had had rheumatic fever four years ago; and repeated epistaxis three years ago. He had now been feeling weak and out of health for six months past, and he had become very pale. During the last two months he had suffered from hæmoptysis, hæmatemesis, and epistaxis—all slight. He had never been jaundiced. On admission to hospital his temperature was 98.8° F., pulse-rate 80, and respiration-rate 30. The cardiac impulse was in the fifth left intercostal space half an inch external to the nipple line, and there was a well-marked apical systolic bruit. The urine contained a little albumen, but no cases. Upon abdominal examination there was a fulness above the umbilicus, and the left half of the epigastric angle was larger than the right. A tumour could be felt in the epigastric and left hypochondriac regions extending below the umbilicus and round into the left lumbar region. It felt smooth and solid, and it reached two inches to the right of the middle line above the umbilicus. The right border was relatively sharp and well-defined. The mass moved downwards with inspiration. It was dull on percussion, and the dulness extended upwards to the fourth left space. The stomach was inflated with gas for diagnostic purposes, and resonance then extended downwards in front of the tumour about two inches.

The blood-count was as follows:—

| | |
|---|---|
| Red blood corpuscles ... | 5,400,000 per cub. mm. |
| White corpuscles ... ... | 2,800 per cub. mm. |
| Hæmoglobin ... ... | 60 p. c. of normal |
| Colour index ... ... | 0.555 |

The differential leucocyte-count was as follows:—

| | |
|---|---|
| Small lymphocytes ... ... | 29 per cent. |
| Large hyaline lymphocytes ... ... | 6 per cent. |
| Polymorphonuclear cells ... ... | 65 per cent. |
| Coarsely granular eosinophile cells ... | none seen |

In view of the apparent relation of the stomach to the tumour, pancreatic cyst seemed not unlikely, and an exploratory operation was performed. The tumour was found to be an enlarged spleen. The lower edge was rounded and thick, and there was extensive capsulitis, so that the notches were obscured. After the operation, and on considering the result of the blood examination and the clinical history, a diagnosis of splenic anæmia was made, with a little doubt as to whether there might not be infective endocarditis.

The explanation of the extension of the tumour so far across the middle line, and so much above the umbilicus, was that some dislocation of the organ had occurred with a considerable degree of rotation and tilting, so that the long axis was lying transversely.

*(To be continued.)*

